# Microfluidic Channels Fabrication Based on Underwater Superpolymphobic Microgrooves Produced by Femtosecond Laser Direct Writing

**DOI:** 10.1021/acsapm.9b00269

**Published:** 2019-09-25

**Authors:** Jiale Yong, Zhibing Zhan, Subhash C Singh, Feng Chen, Chunlei Guo

**Affiliations:** † The Institute of Optics, University of Rochester, Rochester, New York 14627, United States; ‡ Shaanxi Key Laboratory of Photonics Technology for Information, School of Electronics & Information Engineering, Xi’an Jiaotong University, Xi’an 710049, P. R. China

**Keywords:** underwater superpolymphobicity, microfluidic channels, microfluidic systems, femtosecond laser, PDMS

## Abstract

A strategy is proposed here to fabricate microfluidic channels based on underwater superpolymphobic microgrooves with nanoscale rough surface structure on glass surface produced by femtosecond (fs) laser processing. The fs laser-induced micro/nanostructure on glass surface can repel liquid polydimethylsiloxane (PDMS) underwater, with the contact angle (CA) of 155.5 ± 2.5° and CA hysteresis of 2.7 ± 1.5° to a liquid PDMS droplet. Such a phenomenon is defined as the underwater “superpolymphobicity”. Microchannels as well as microfluidic systems are easily prepared and formed between the underwater superpolymphobic microgroove-textured glass substrate and the cured PDMS layer. Because the tracks of the laser scanning lines are programmable, arbitrary-shaped microchannels and complex microfluidic systems can be potentially designed and prepared through fs laser direct writing technology. The concept of “underwater superpolymphobicity” presented here offers us a new strategy for selectively avoiding the adhesion at the polymer/substrate interface and controlling the shape of cured polymers; none of these applications can find analogues in previously reported superwetting materials.

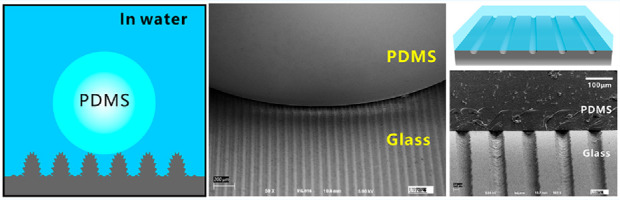

## 1 INTRODUCTION

Microfluidic systems, one of the most important components in the lab-on-chip devices, have attracted increasing interest due to their important applications in analytical chemistry, biomedical assay, and chemical and biochemical syntheses.^1−8^ In contrast to conventional large-scale facilities, microfluidic devices have many distinct advantages in chemical and biomedical applications, including low volume of sample consumption, faster analytical processing, and increased precision and accuracy.^3,5,9,10^ Served as the key components of microfluidic devices, closed microchannels are mostly fabricated by conventional lithography-based techniques,^4,11^ e.g., soft lithography^12^ and imprint lithography.^13,14^ However, the fabrication of microfluidic channels based on the lithography techniques is complex, time-consuming, and expensive; it also typically requires a clean room, specialized equipment, advanced training, and expensive reagents.^15−17^ Developing a simple strategy for preparing microfluidic channels is significant and remains a major challenge.

A femtosecond (fs) laser has been used to achieve various superwettabilities on a solid surface.^18−24^ Here, we find that the fs laser-induced microstructures on the glass surface show excellent underwater superpolymphobicity. The laser-treated surfaces can repel liquid polydimethylsiloxane (PDMS) under-water and reduce the adhesion between the glass substrate and the liquid PDMS. On the basis of the underwater super-polymphobicity of the resultant microgrooves with nanoscale structure, we propose a simple strategy to fabricate microfluidic channels between the glass surface and the cured PDMS coating. The size of the microchannels can be designed by laser power during laser scanning.

## 2 RESULTS AND DISCUSSION

**Figure 1:**
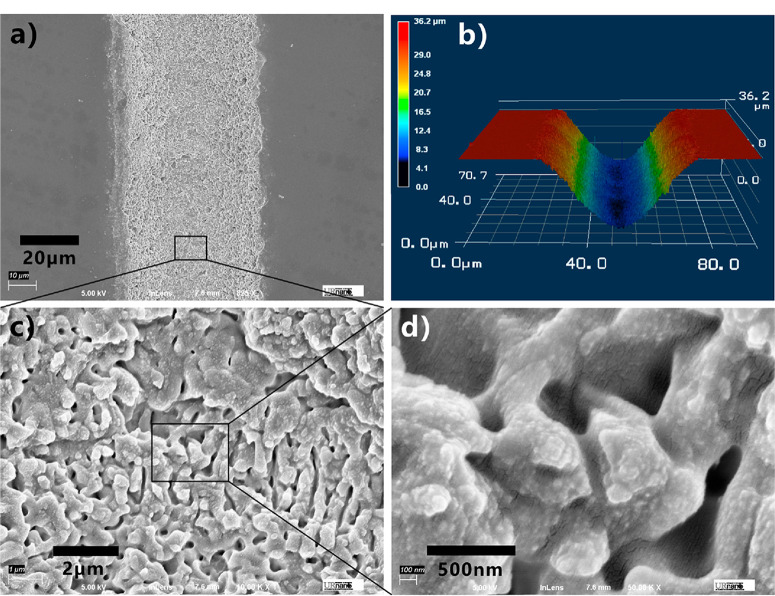
Morphology of the laser-induced microgroove. (a) Scanning electron microscopy (SEM) image of a single groove. (b) 3D profile of the groove. (c, d) SEM images of the bottom surface of the groove.

Femtosecond laser processing is an important tool in precision manufacturing because of its extremely short pulse width and ultrahigh peak intensity.^25−28^ This technology can directly create a series of micro-and nanostructures on different material surfaces and then achieve various special surface wettabilities.^29−34^ Laser scanning can generate a groove on the sample surface. Figure 1 exhibits the surface microstructure of the laser-induced groove on the glass surface. The glass was treated by laser at the power of 300 mW. The unablated area is flat, while the laser-ablated area is characterized by a microgroove with nanoscale rough surface structure (Figure 1a). The groove is about 53.0 μm in width and 21.4 μm in depth (Figure 1b). There are abundant protrusions and holes with the size of hundred nanometers randomly coating on the bottom of the microgroove (Figure 1c). The surfaces of the nanoprotrusions are embellished with fine pimple-like nanostructures with the size of tens of nanometers (Figure 1d).

The formation of the microgroove and the inner nanostructure on glass surface produced by fs laser pulses can be explained via hot melt theory.^31,32,35^ Once the fs laser pulses irradiated the glass surface, the nonlinear absorption of glass surface will trigger the avalanche ionization. The energy of pulses is partly absorbed by glass surface (mainly absorbed by the electron) and results in a thin melt layer over the substrate. Instantaneously, the energy is transferred from electron to crustal lattice and further diffuses into glass substrate. Meanwhile, the ionized material is removed away and ejected with the expansion of the high-pressure plasma, which causes a permanent damage of glass surface. The intensity of the fs laser pulses is extremely high and at the Gauss-curved distribution. Strong ablation and material removal occur at the laser-focused spot because the laser fluence is highest at the spot center. As a result, microgroove is created in the center of the laser scanning line. Generally, a submicrometer structure can be generated by low-intensity fs laser pulses. With moving the spot center forward, the spot fringe with low fluence will irradiate the glass surface; thereby, a nanostructure further forms on the inner surface of the microgroove. In addition, the ejected nanoscale particles at the molten state that fall back to the substrate and resolidify can also lead to the coating of fine nanostructures on the microgroove surface.

**Figure 2:**
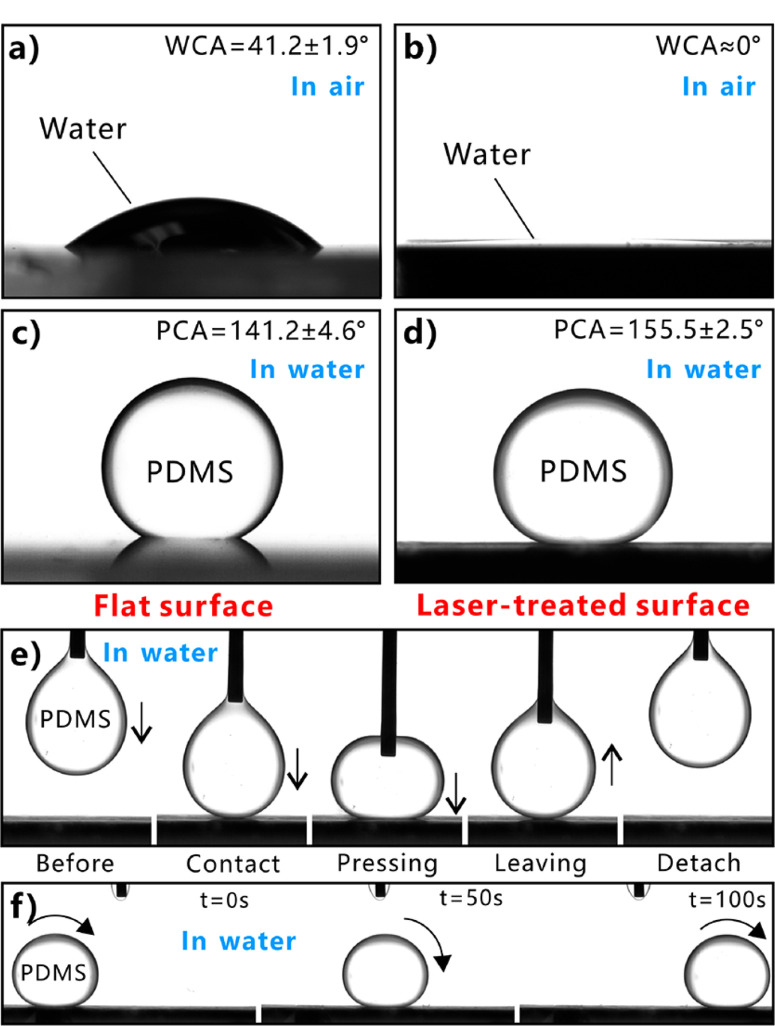
Wettability of glass surface. (a, b) Water droplet on the (a) untreated flat glass and (b) laser-treated glass in air. (c, d) Liquid PDMS droplet on the (c) untreated flat glass and (d) laser-treated glass in water. (e) Underwater liquid PDMS droplet being moved to touch and then leave the laser-treated glass surface. (f) Underwater liquid PDMS droplet rolling off the laser-treated surface.

To investigate the wettability of the laser-induced structure,we ablated a glass surface at a small interval (Λ) of the scanning lines, e.g., Λ = 40 μm. In this case, the laser-induced microgrooves overlap, so that the whole surface of the glass substrate is uniformly covered with nanostructure which is the same as the bottom of a single groove (Figure 1c,d). The flat glass surface is inherently hydrophilic, with a water contact angle (WCA) of 41.2 ± 1.9° to a water droplet (Figure 2a). The hydrophilicity can be amplified to superhydrophilicity by the laser-textured nanostructure. If a water droplet touched the laser-structured glass, the droplet would quickly spread out on the glass surface (Movie S1, Supporting Information). The measured WCA is approximately equal to 0° (Figure 2b). With regard to the underwater liquid PDMS droplet, the untreated flat sample surface shows underwater polymphobicity with a polymer contact angle (PCA) of 141.2 ± 4.6° (Figure 2c). The surface also shows high adhesion to the PDMS droplet in water because the contact angle hysteresis (CAH) of an underwater liquid PDMS droplet on the untreated glass is measured to be 88.3 ± 8.6°. In contrast, liquid PDMS droplet can maintain a spherical shape on the laser-structured glass surface in water (Figure 2d). The PCA reaches up to 155.5 ± 2.5°. Figure 2e and Movie S2 show the process of an underwater liquid PDMS droplet being moved to touch and then leave the laser-treated surface. No obvious shape deformation of the PDMS droplet was noticed at the moment of just leaving the sample surface, even though the droplet was strongly pressed toward the sample surface. Such a result demonstrates the adhesion between the laser-induced microstructure and under-water liquid PDMS is very low. As long as the sample was tilted to 2.7 ± 1.5°, the PDMS droplet on the textured glass surface was able to easily roll off although the rolling speed is low (Figure 2f and Movie S3). The CAH is measured to be only 2.7 ± 1.5°. Therefore, the laser-induced nanostructures have great repellence to liquid PDMS in a water medium. The phenomenon that a liquid polymer droplet has a contact angle higher than 150° on a substrate and the substrare also shows extremely low adhesion to the polymer droplet in water can be defined as underwater “superpolymphobicity”, following the etymology of the term “superhydrophobicity” (“polym” is usually short for “polymer”, “hydro” is from the Greek word for “water”, and “phobic” is from the Greek word for “fear”).^36,37^ Consequently, laser treatment endows glass surface with not only rough surface structure but also superpolymphobicity to underwater liquid PDMS droplets.

**Figure 3:**
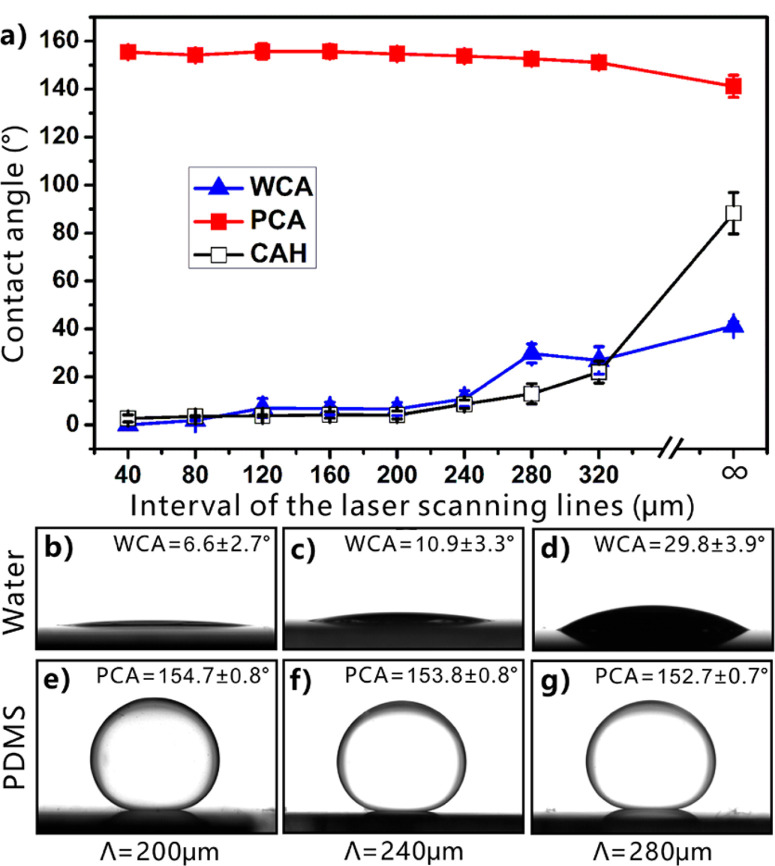
(a) Influence of Λ on the surface wettability of the laser-treated glass substrates. (b−d) Shapes of an in-air water droplet on the structured glass surfaces with different Λ. (e−g) Shapes of a PDMS droplet on the glass surfaces with different Λ in water.

The interval (Λ) of scanning lines is a crucial parameter in laser processing. Figure 3a depicts the relationship between the Λ and the wettability of laser-treated glass surface. For the Λ being <200 μm, the surfaces exhibit excellent in-air superhydrophilicity and underwater superpolymphobicity. All the WCA values are <10°. With regard to the underwater polymer wettability, the PCA values are all higher than 150°. In addition, the CAH values are smaller than 10°. With the Λ increasing from 200 to 240 μm and then to 280 μm, the WCA increases from 6.6 ± 2.7° (Figure 3b) to 10.9 ± 3.3° (Figure 3c) and then to 29.8 ± 3.9° (Figure 3d), so the water wettability changes from superhydrophilicity to ordinary hydrophilicity. The PCA values do not have an obvious decline and keep larger than 150° (Figure 3e−g). However, the CAH values increases from 4.1 ± 1.7° (Λ = 200 μm) to 8.6 ± 1.8° (Λ = 240 μm) and then to 13.0 ± 4.2° (Λ = 280 μm), indicating that the adhesion between the laser-treated substrate and the PDMS droplet increases from ultralow to high (Figure 3a). Therefore, the superhydrophilicity and underwater superpolymphobicity with low adhesion can be obtained at Λ ≤ 200 μm.

**Figure 4:**
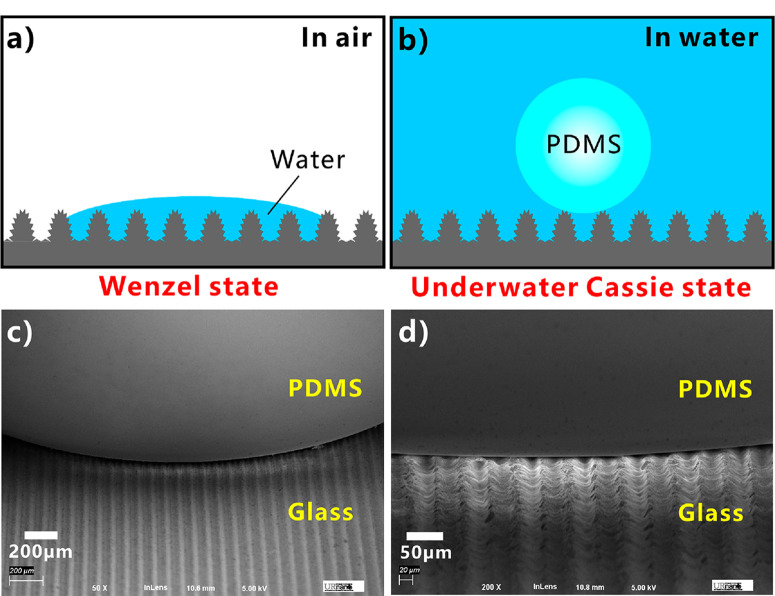
Formation mechanism of the underwater superpolymphobicity. (a) Contact between a water droplet and the laser-induced microstructure in air: Wenzel wetting state. (b) Contact between a liquid PDMS droplet and the laser-induced microstructure in water: Underwater Cassie wetting state. (c, d) SEM images of the contact between the PDMS droplet and the glass microgrooves. The SEM images were captured after curing the PDMS droplet and removing the water environment.

The underwater superpolymphobicity of the fs laser-ablated surface can be explained based on the wetting state between sample surface and underwater polymer droplet. After fs laser treatment, rough nanostructure forms on the glass surface. The rough nanostructure can amplify the natural wettability of the glass because the actual surface area is greatly increased by the laser-induced nanostructure, making the glass surface change from intrinsic hydrophilicity to superhydrophilicity. Water on the textured surface is at the Wenzel state, so the surface nanostructures are completely wetted by water (Figure 4a).^36,38^ When the sample is immersed in water, water will fill in and occupy all space between the nanostructures, like being trapped among the rough structures. As a liquid PDMS droplet is dropped on the laser-structured surface, the droplet will reside on a composite water/solid interface, generating a PDMS/solid/ water three-phase system, as shown in Figure 4b. The water that is trapped in surface nanostructure provides a repulsive force to the liquid PDMS droplet based on the repulsive interaction between nonpolar (PDMS) and polar (water) molecules and the insolubility between liquid PDMS and water. The contact between the PDMS droplet and the nanostructure is remarkably prevented by the trapped water cushion. Figure 4c,d shows the real contact situation between the PDMS droplet and the nanostructures of glass surface. The SEM images were captured after curing the PDMS droplet and removing water environment. It is demonstrated that the PDMS droplet only touches the top part of the surface nanostructures. The PDMS droplet is repelled by the laser-induced nanostructures in water. There-fore, the contact model between the underwater PDMS droplet and the sample surface is at the Cassie wetting state (an underwater version) (Figure 4b), so the laser-treated glass surfaces show excellent underwater superpolymphobicity.^29,36,38^ Although the underwater superpolymphobicity on the laser-induced microstructure and underwater superoleophobicity have similar formation mechanisms, the underwater super-polymphobicity is very different from the superoleophobicity because the surface repels polymers rather than oils and they have different applications field.^36,39−41^ For example, the underwater superpolymphobic microstructure has the applications in avoiding the adhesion between the polymer/substrate interface and designing the shape of cured polymers; none of these applications can find analogues in previously reported superwetting materials.^42−45^

**Figure 5:**
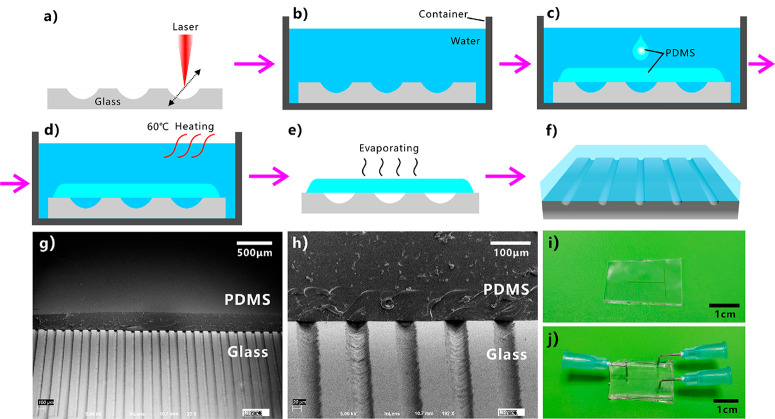
Preparation of microfluidic channels between glass substrate and cured PDMS based on the laser-induced underwater superpolymphobic microgrooves. (a−f) Schematic of the preparation process: (a) formation of microgrooves on glass surface by fs laser direct writing, (b) immersion of the glass substrate into a water container, (c) pouring the uncured liquid PDMS onto the glass substrate, (d) curing the PDMS layer at 60 °C, (e) taking the glass/PDMS sample out of water and removing the adhered water, and (f) the microchannels. (g, h) Cross-sectional SEM images of the resultant microchannels between glass substrate and PDMS coating. (i) T-shaped microgroove on a glass surface. (j) A simple microfluidic system fabricated by using the laser-induced underwater superpolymphobic T-shaped microgroove.

On the basis of the liquid PDMS repellence of laser-induced microgrooves, we propose a strategy to prepare microchannels between glass substrate and PDMS. As shown in Figure 5a, separated microgrooves were first written on a glass substrate by fs laser scanning. The track of the microgrooves can be arbitrarily designed by control program during laser treatment. Then, the glass substrate with microgrooves was put in a container filled with water (Figure 5b). The superhydrophilicity of the laser-induced microstructure allowed the glass surface to be completely wetted by water. Subsequently, the uncured liquid PDMS was poured onto the glass surface in water (Figure 5c).

The liquid PDMS formed a thin layer on the glass substrate. Meanwhile, the liquid PDMScould only touch the nonablated flat glass domain but was repelled by the microgrooves due to the underwater superpolymphobicity. The contact between liquid PDMS and microgrooves was prevented by the laser-induced superpolymphobic microstructure. The liquid PDMS was unable to enter into the microgrooves and was lifted by two edges of the microgrooves. As the liquid PDMS layer was cured by storing the water container at 60 °C for 3 h, a solid PDMS film adhering to the glass substrate formed (Figure 5d). Finally, we took the glass/PDMS sample out of water container and made the adhered water completely evaporate (Figure 5e). As a result, hollow microchannels were obtained between the laser-induced microgrooves and the cured/solidified PDMS film (Figure 5f−h). The microchannels can be further applied to form various microfluidic systems. As a simplest example, Figure 5i,j shows the fabrication of a T-shaped microfluidic system. A T-shaped microgroove was first written on a glass substrate by a fs laser (Figure 5i). Then, a PDMS layer was coated over the microgroove through the above-mentioned method. After jointing the external pipes, a simple T-shaped microfluidic system was obtained (Figure 5j). Because the track of laser scanning line is programmable, so we can design arbitrarily shaped microchannels and potentially fabricate complex microfluidic systems by fs laser direct writing technology.

**Figure 6:**
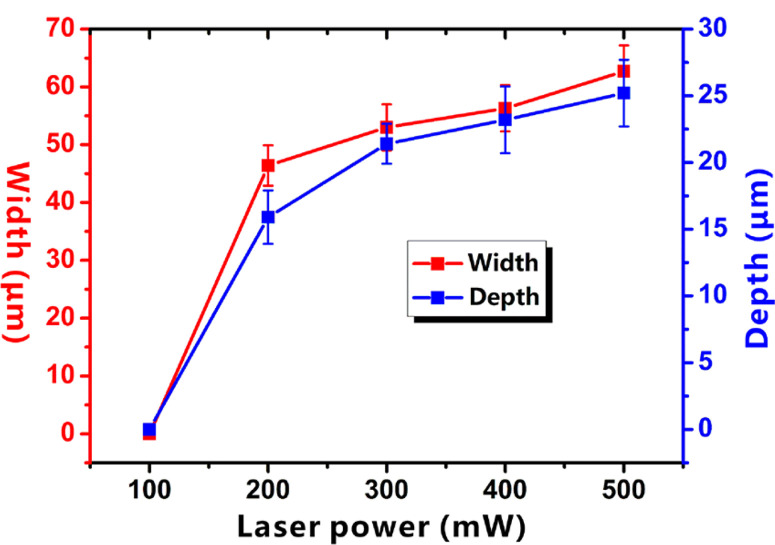
Relationship between the width/depth of the microgrooves and the laser power.

The size of the resultant microchannels is determined by the width and depth of the laser-structured microgrooves, which can be designed by laser power during the laser processing. Figure 6 depicts the influence of the laser power on the width and the depth of resultant microgrooves. It is found that both the width and the depth increase with the increase of used laser power. In addition, the width and the depth have a similar variation trend. Therefore, higher laser power results in a larger microfluidic channel.

## 3 CONCLUSIONS

In conclusion, we demonstrate a simple strategy to fabricate microfluidic channels based on the underwater superpolymphobicity of fs laser-structured microgrooves. The fs laser can directly write microgrooves with nanoscale surface structures on the glass substrate. Such micro/nanostructures have the great ability to repel liquid PDMS in water. An underwater liquid PDMS droplet has a PCA of 155.5 ± 2.5° and CAH of 2.7 ± 1.5° on the laser-ablated surface, indicating remarkable underwater superpolymphobicity. Based on the underwater superpolymphobicity of the laser-induced microgrooves, microchannels are easily prepared between glass substrate and PDMS layer. The microchannels can be further applied in various microfluidic systems. The track of the microchannels can be programmatically designed, so arbitrarily shaped microchannels, as well as complex microfluidic systems, can be potentially prepared by fs laser direct writing technology.

The concept of “underwater superpolymphobicity” also offers us a potential strategy for controlling the shape of cured polymer and preventing the adhesion between polymer and solid substrate. Therefore, the underwater superpolymphobic micro-structures can be applied in nearly all the applications based on the polymer materials, such as polymer preparation, polymer casting industry, and three-dimensional (3D) printing technology.

## 4 EXPERIMENTAL SECTION

**Materials.** The glass sheets are microscope slides (Fisherbrand). The liquid PDMS (DC-184, Dow Corning Corporation) is the mixture of the prepolymer and the curing agent with the volume rate of 10:1.

**Femtosecond Laser Treatment**. The glass sheet was fixed on a moveable platform. By use of a plano-convex lens with the focal length of 25 cm, the 67 fs laser beam with the center wavelength of 800 nm and repetition rate of 1 kHz was focused on the glass surface. The laser scanning speed was set constant at 2.5 mm s^−1^.

**Fabrication of Microchannels**. Microgrooves were first written on a glass surface by fs laser scanning. Then, the sample was immersed in a water container. Uncured liquid PDMS was further poured onto the glass surface in water. After forming a PDMS thin layer above the glass surface, the water container was stored at 60 °C for 3 h to cure the liquid PDMS. Next, the glass/PDMS sample was taken out of water container. As the adhered water completely evaporated, hollow microchannels were obtained between the laser-induced microgrooves and the cured/ solidified PDMS film.

**Characterization**. The laser-induced surface microstructure on glass surface was observed by a S-4100 scanning electron microscope (Hitachi, Japan) and a VK-9700 laser confocal microscope (Keyence, Japan). The wettability (including contact angle, sliding angle, and contact angle hysteresis) of the sample surfaces was determined by a SL2000KB contact-angle measurement (Kino, USA). Because the densities of water and liquid PDMS are very close, the adopted water environment in this experiment is a mixture of water and ethanol (v:v = 1:1), enabling liquid PDMS droplet to be easily dispensed onto the sample surface.

## ASSOCIATED CONTENT

### Supporting Information

Movie S1: a small water droplet fully wetting the laser-treated glass surface in air (MP4)

Movie S2: a liquid PDMS droplet being moved to touch and then leave the laser-treated surface in water (MP4)

Movie S3: a liquid PDMS droplet rolling off the laser-treated surface in water (MP4)
